# Microstrip Antenna Development for Radar Sensor

**DOI:** 10.3390/s23020909

**Published:** 2023-01-12

**Authors:** Lajos Nagy

**Affiliations:** Department of Broadband Infocommunications and Electromagnetic Theory, Budapest University of Technology and Economics, Műegyetem rkp 3, H-1111 Budapest, Hungary; nagy.lajos@vik.bme.hu

**Keywords:** microstrip antenna, level measurement, contactless measurements

## Abstract

Tank level measurement is an important research area in radar technology. Level measurement using radar technology is a safe solution even under extreme process conditions, such as high pressure, temperature, and vapors. Special antennas are required to meet electromagnetic requirements such as high gain, low sidelobe level, and high bandwidth. Another requirement is a small size and good manufacturability of the antenna. One promising solution is the use of microstrip antennas for tank-level measurements. This paper presents a special microstrip antenna, that meets the main requirements in addition to a circular antenna layout, which fills optimally the tank hatch. To do this, we created a special antenna layout with a suitable feeding network and added an optimized ring around the antenna system, which significantly reduces the sidelobe level. The center frequency of the antenna is 25 GHz with a 1 GHz bandwidth.

## 1. Introduction

Constant product quality, operational safety, and economic efficiency can only be ensured by continuous measurements and intervention and control systems based on these measurements. Liquids, pastes, bulk solids, and liquefied gases are most often stored in tanks, silos, or mobile containers. These tanks are used in the chemical and petrochemical industries, the pharmaceutical and life sciences industries, the water industry, the chemical and petrochemical industries, the water and wastewater, and the food and energy industries.

There are several classical and modern methods for measuring the product level in process and storage tanks. Applications are in the chemical, petrochemical, pharmaceutical, water, and food industries, mobile tanks on vehicles and ships, and natural reservoirs such as seas, dams, lakes, and oceans. Typical tank heights range from 0.5 m to 37 m. [1,2]

Two main measurement tasks can be distinguished [3]:

- continuous level measurement, i.e., level indication,
- level detection, i.e., detection of an alarm limit to prevent overfilling.

**Figure 1:**
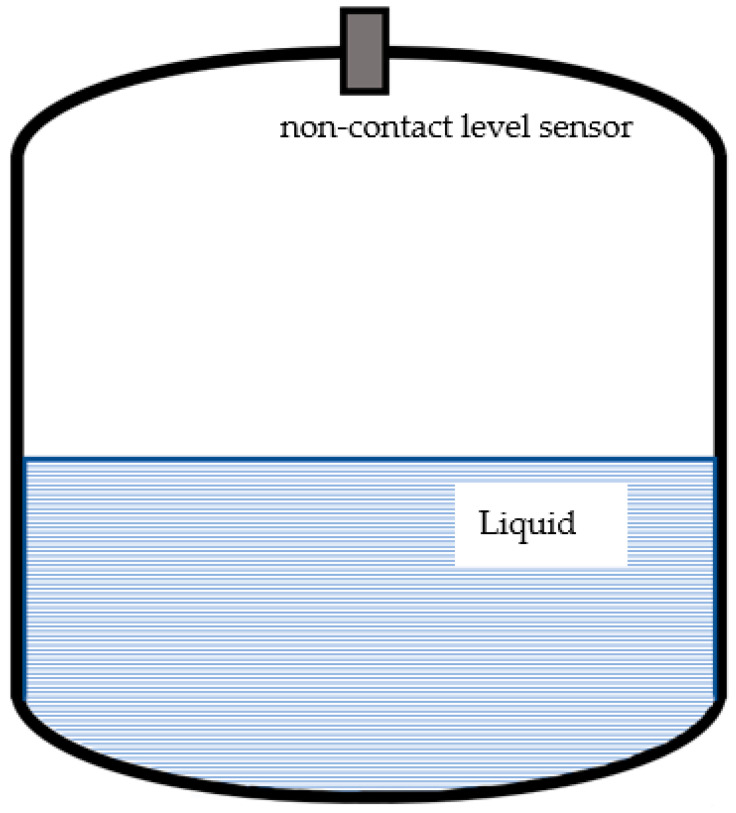
Tank with liquid and non-contact sensors on the top of the tank.

Many level devices are mounted on top of the tank and measure primarily the distance between their mounting position and the product’s surface [3] (Figure 1).

For level measurement, a significant number of different principles measurement techniques are available [4], and it is advisable to select the optimum technique and sensor. The commonly used tank-level measurement methods are based on the next basic principles: buoyant object floats, RF capacitance, radar, ultrasonic, and hydrostatic head/tank gauging. No single principle applies to all measurement areas. Therefore, measurement systems should be selected based on what works reliably under the given conditions, and at the same time meet the accuracy of the measurement.

We developed a sensor antenna for radar-level pulse [5] measurement, which is based on the principle that the time required for the propagation of microwaves. It is the time takes for the wave packet to travel during the entire round trip between the non-contact transducer detected material level and the measuring device. Pulse radar has been widely used for distance measurement since the early days of radar. Radar level measurement is a safe non-contact solution even under extreme process conditions, at high pressure, and temperature, vapors. For radar measurements, special antennas are required to meet electromagnetic requirements such as high gain, low sidelobe level, and high bandwidth. Another requirement is a small size and good manufacturability of the antenna.

A promising solution is the use of microstrip antennas [6,7,8,9] for contactless tank level measurement. Its main advantages are the small height and weight, and easy manufacturability.

Many other antenna types are promising for tank-level radar measurements, such as conical horn antennas [10], parabolic reflector antennas [11], and dielectric antennas [12,13,14].

Recently, theoretical work is conducted applying metamaterial-based antennas [15] also for tank-level measuring sensor antennas. These analytical or numerical solutions allow for the designing and building of useful antennas and devices; however, more effective fabrication techniques need to be developed for these devices. Another difficulty of the metamaterial type antennas is the bandwidth because these devices show generally narrowband behavior and bandwidth is not analyzed [15].

In this paper, a special microstrip antenna was designed, that meets the main requirements, i.e., high gain, low sidelobe level, high bandwidth, small size, and good manufacturability of the antenna. The antenna was realized on a low-loss RT5880 substrate. As opposed to the traditional N × N microstrip antenna layout, we used a special 24-element layout. With this arrangement, the side beam suppression has been significantly reduced. The feeding network of the 24-element antenna system is made up of three main power-divider elements, with three different power-division ratios. CST Microwave Studio was used to simulate and fine-tune the antenna array. The measurements were performed in an anechoic chamber, where we measured input reflection and antenna radiation patterns in the 23–27 GHz frequency range.

## 2. Antenna Design

The special microstrip antenna satisfies two main requirements, namely a circular antenna layout and an optimized ring around the antenna system, which significantly reduces the sidelobe level. The center frequency of the antenna is 25 GHz with a bandwidth of 1 GHz.

In this chapter, the design of the feeding network for a microstrip antenna system with a 24-element circular confinement geometry is presented in detail as the optimization of the sideband suppression ring. The electromagnetic design of the antenna was carried out using the Ansys Designer 2D planar [16] and CST Microwave Studio 3D [17] software.

**Figure 2:**
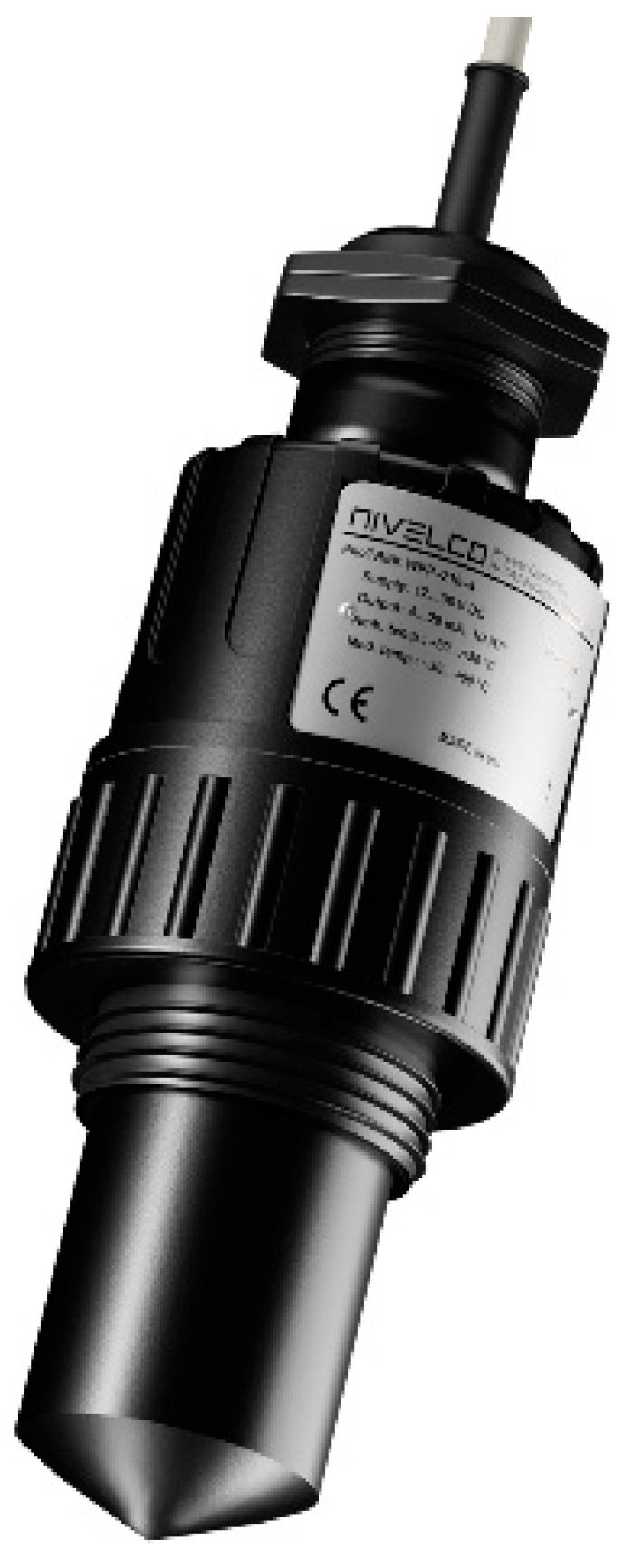
Standard level meter house.

To fulfill the requirements an antenna system will be introduced, which optimally fills the standard circular opening of the tanks. The sizes of the antenna designed were chosen to fit into and optimally fill the standard circular opening of the tanks (Figure 2).

**Figure 3:**
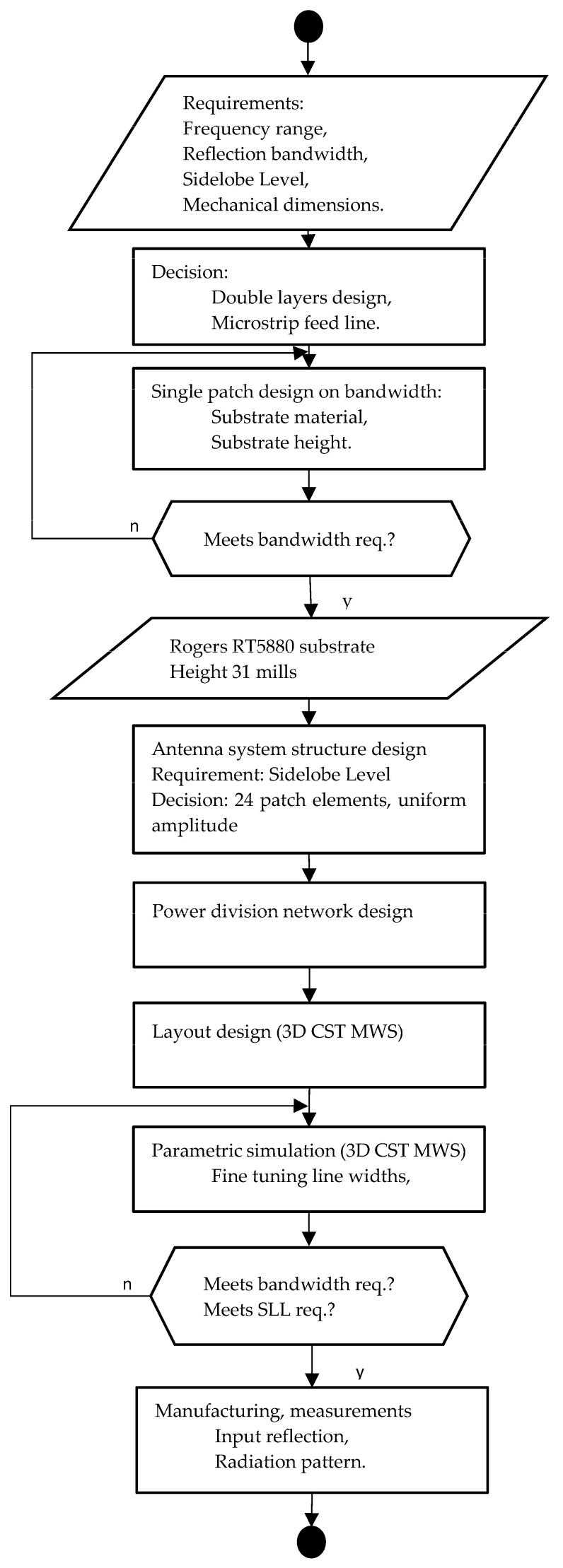
Flowchart of the antenna overall design procedure.

The feeding network is designed for the antenna system for optimal illumination. We introduce the detailed design process of the microstrip antenna element, placing, and feeding network. (Figure 3)

### 2.1. Single Radiator—Microstrip Patch

With the development of modern wireless communications and radar technology, there is an increasing demand for compact antennas. Microstrip Patch Antennas (MPA) are lightweight, low cost, and easy to integrate into microwave circuits, and are therefore widely used in mobile and wireless applications. The use of MPAs in mobile wireless communication systems allows the implementation of small, low antenna sizes [18,19].

The basic geometry of the antenna is a dielectric substrate located between the ground plane and the radiating patch. The size and shape of the MPA radiator, as well as the height and the type of substrate material, are the most important design parameters for evaluating the performance of an MPA. The substrate, in addition to providing mechanical strength to the entire antenna, also allows the propagation of surface waves. The dielectric substrate layer separates the radiator from the ground, its material and thickness are of primary importance for the antenna bandwidth. Since the antenna dimensions are finite, the fields along the antenna are finite, and the fields along the edges are frictional [19].

**Figure 4:**
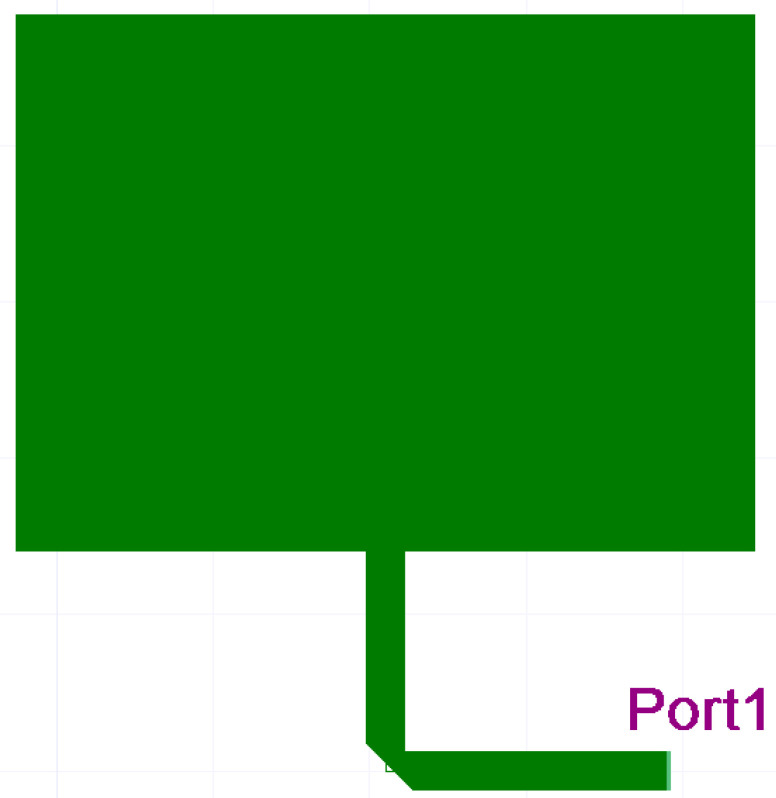
Single radiator Microstrip Planar Antenna (Ansys Designer model).

Several authors have investigated the effect of substrate thickness and material on the bandwidth of MSA. We simulated a single MPA (Figure 4) to test its relative bandwidth and radiation efficiency for three substrates and five heights of substrates [20,21,22,23,24,25,26,27,28].

The substrates investigated were Rogers RT5880, RO4003, and RO3010 with substrate heights of 5, 10, 20, 31, and 62 mils. The simulation results are summarized in Table 1.

Table 1 clearly shows that antenna bandwidth can be increased by increasing the substrate height but the efficiency of the antenna changes in the opposite direction. The goal of 1 GHz bandwidth (4%) can be achieved as Table 1 shows. The candidate is an RT5880 substrate with either 20 or 31 mils of height for the best radiation efficiency. Finally, the following standard 31mil substrate thickness was chosen to compensate for bandwidth loss due to manufacturing errors of antennas and feeding networks.

**Figure 5:**
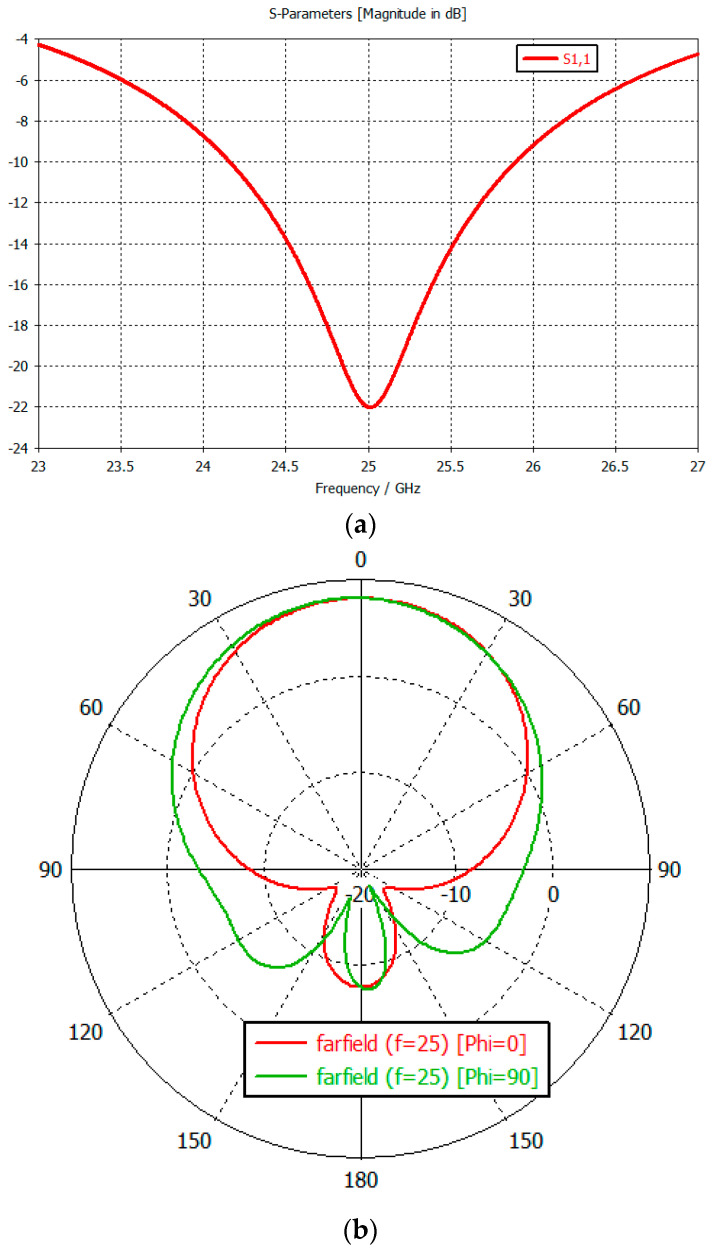
(**a**) Single radiator Microstrip Planar Antenna input reflection. (**b**) Single radiator Microstrip Planar Antenna radiation pattern.

The single MPA antenna, simulated on 31mils RT5880 substrate, input reflection, and radiation pattern are shown in Figure 5.

The single radiator has 8 dB gain at 25 GHz and 80.7° and 72.7° beamwidth in E and H planes (Figure 5b).

The next design step is the radiator array structure design.

### 2.2. The Radiator Array Structure

**Figure 6:**
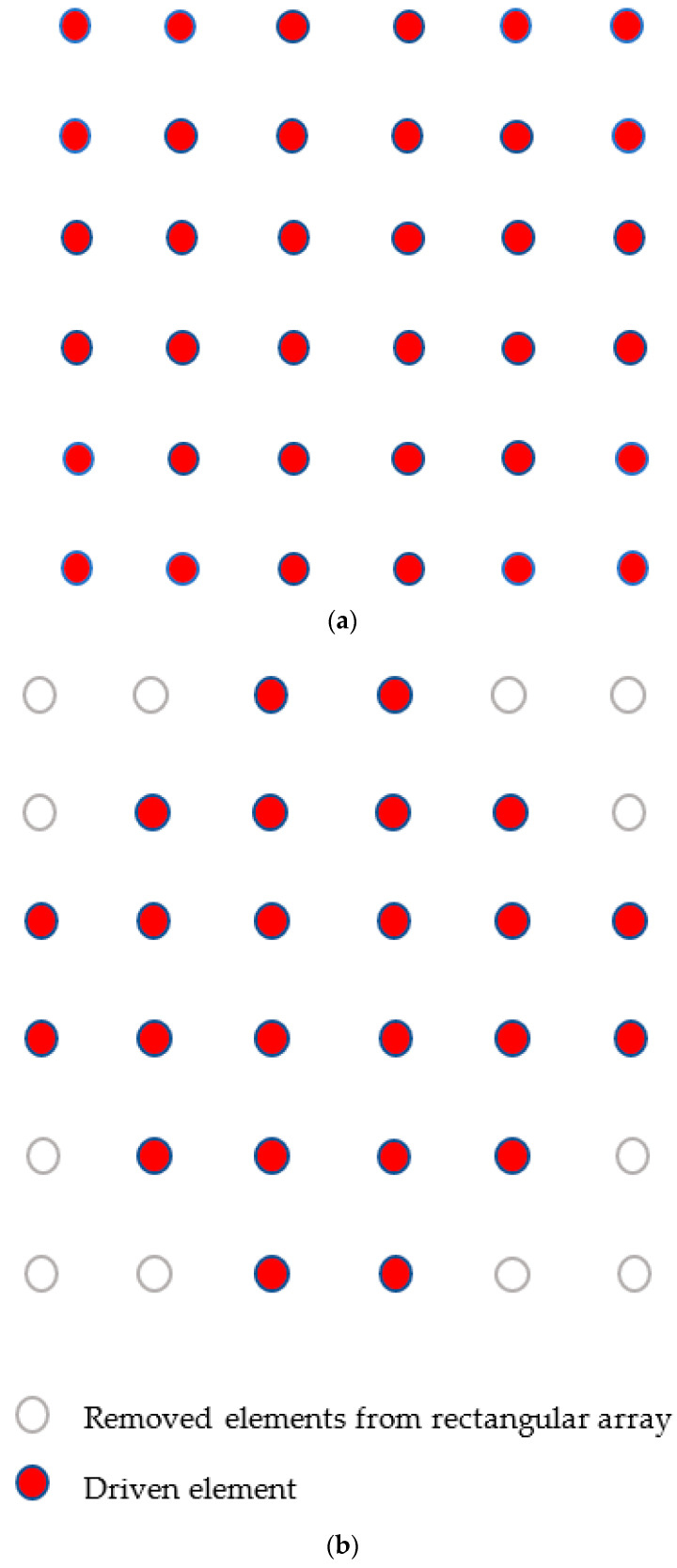
(**a**) A 6 × 6 planar uniform rectangular array. (**b**) 24-element array.

The excitation of each radiator, which can be of uniform or non-uniform amplitude, is adjusted by the feeding network. We first examined a 6 × 6 planar array of uniform rectangular shape and a special 24-element array. (Figure 6)

**Figure 7:**
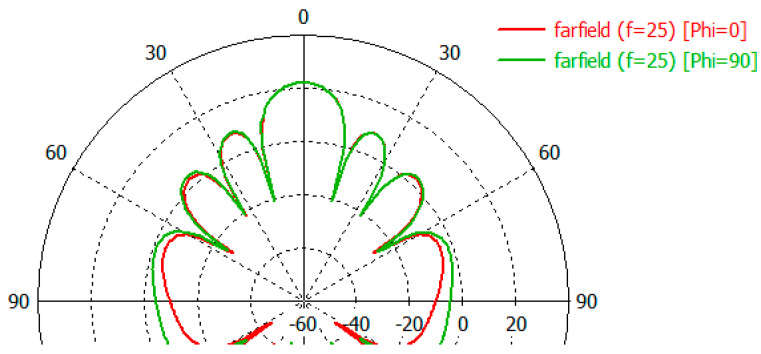
The E and H plane radiation pattern of rectangular 6 × 6 array.

**Figure 8:**
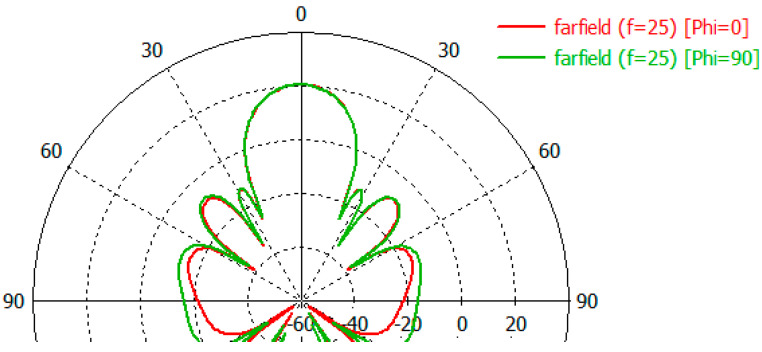
The E and H plane radiation pattern of 24 elements array.

The directional characteristics of the two arrays were compared using the CST array simulator. The radiation pattern for the 6x6 uniform and 24 elements arrays can be seen in Figure 7 and Figure 8.

The main point of comparison was the Sidelobe Level. (Table 2) The 24 elements array has a 2.1dB better SLL.

**Figure 9:**
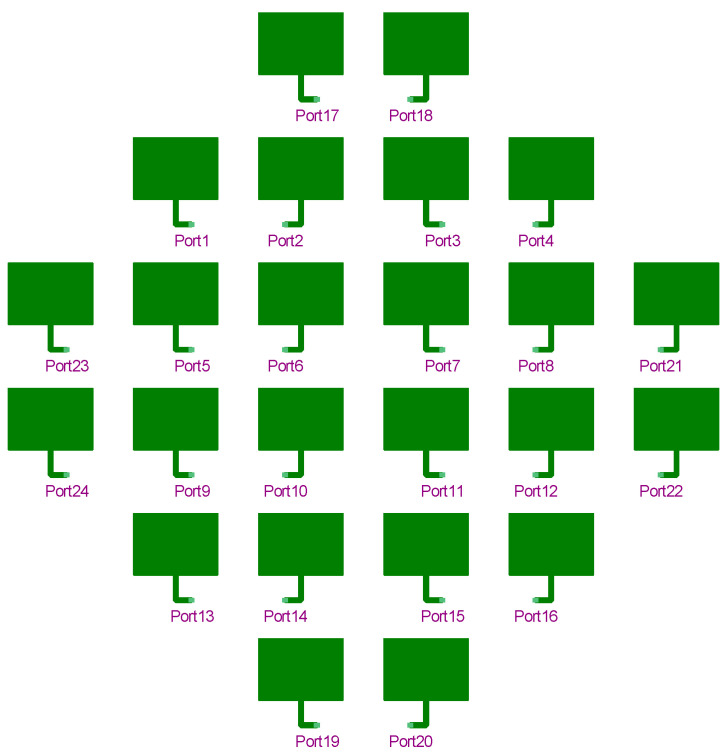
The 24 elements array layout.

The final arrangement of microstrip radiators in the Ansys Designer simulator can be seen in Figure 9.

The antenna-connected transmission lines in Figure 9 have already been arranged for the optimal feeding network.

### 2.3. Feeding Network

The feeding network is made up of three main elements. We implemented three different power division ratios [29].

The design of the power dividers was based on the simple T-junction.

**Figure 10:**
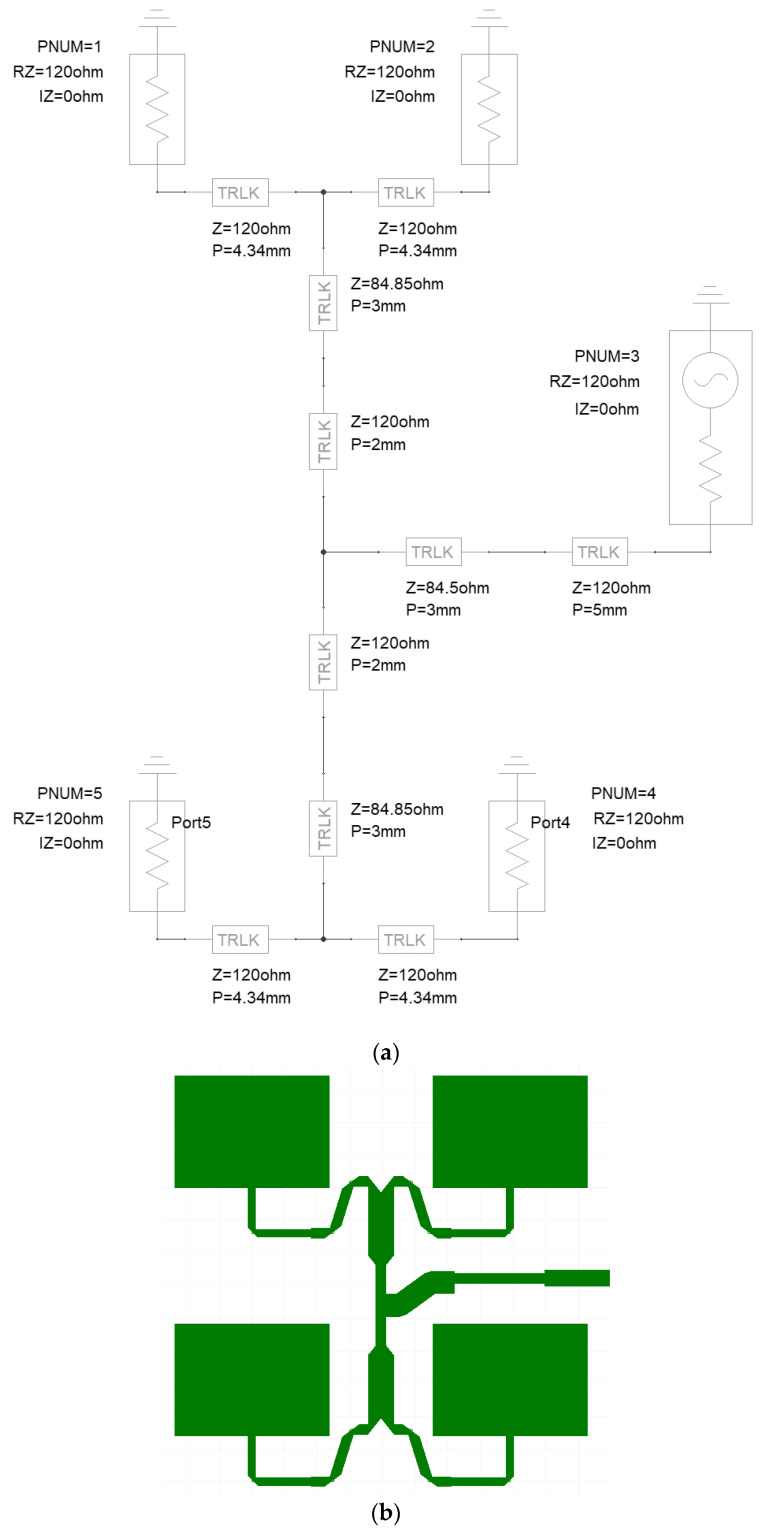
(**a**) Power division network for a 1 to 4 power ratio, with ideal transmission lines. (**b**) Power division network for a 1 to 4 power ratio. Microstrip radiators with microstrip transmission line transformers.

**Figure 11:**
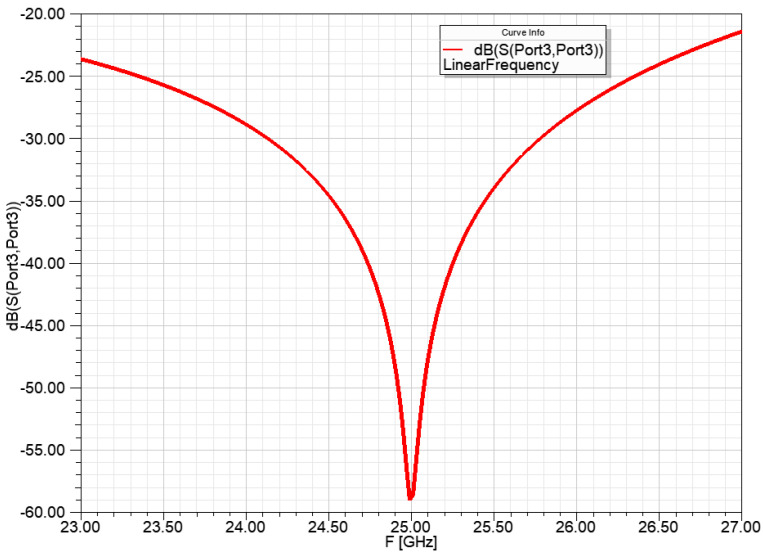
Input reflection of the power division network for a 1 to 4 power ratio, with ideal transmission lines.

**Figure 12:**
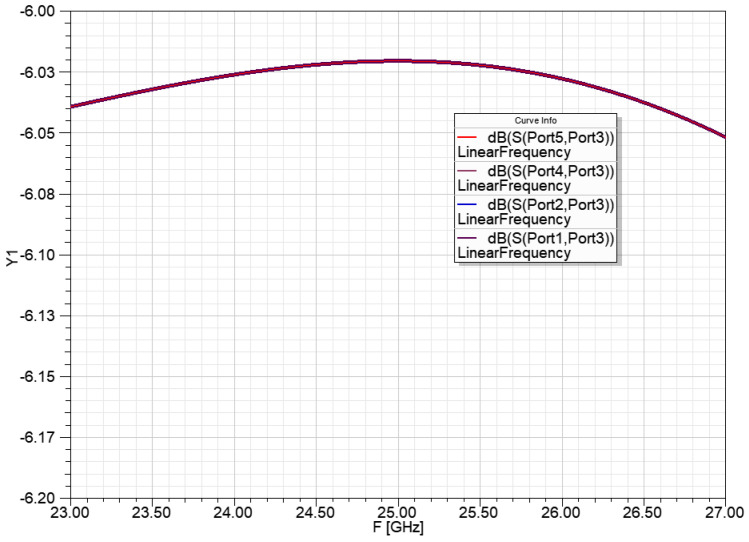
Power ratio for the four radiators of the power division network for 1 to 4 power ratio, with ideal transmission lines.

The power dividers are the following:

Power divider for 1 to 4 power ratio (Figure 10),

3-way power divider from 1 to 4:2:4 power ratio (Figure 11),

4-way power divider from 1 to 2:10:10:2 power ratio (Figure 12).

After calculating the elements of the power dividers, we used Ansys Designer/Nexxim [17] for the final design and analysis of the three main elements of the feeding network. The design was carried out in two steps, first, the circuit model simulator was used to set the matching and power ratio using the ideal transmission line transformers, and then the 2.5D simulator was used to optimize the layout with microstrip transmission lines. Each of the corners was mitered to reduce the reflection.

#### 2.3.1. Power Division Network for the 1 to 4 Power Ratio

The purpose of this network is to feed four identical radiators with the same power and phase. The single microstrip radiator has a 120 ohms input impedance at a center frequency of 25 GHz.

Finally, at the planar feeding network design, a transmission line step was introduced between the microstrip radiator and the T-matching element (Figure 10b).

The resonance frequency of the power division network for the 1 to 4 power ratio was set to 25 GHz (Figure 11).

Figure 12 shows the power ratio for the four radiators of the power division network for the 1 to 4 power ratio. The −6.025 dB power transfer means that the network results in a 1:4 power division with a −0.025 dB loss. Transmission lines in the circuit models are ideal but lossy.

#### 2.3.2. Power Division Network from 1 to 4:2:4 Power Ratio

The upper part of the network feeds 2 × 4 + 2 antennas with the same power. The 2 × 4 feeding part consists of two power division networks for a 1 to 4 power ratio.

The design of the three ways power dividers was based on two equations, the impedance matching equation, and the power ratio equation.

The equation for impedance match:

(1) $$\frac{1}{Z_{in}}=\frac{1}{Z_{1}}+\frac{2}{Z_{2}}$$

where

$Z_{in}$ the divider input impedance (40 ohms),

$Z_{1}$ the impedance of ways 1 and $Z_{2}$ the impedance of way 2.

The power sum equation:

(2) $$P_{in}={\left[I_{1}+2I_{2}\right]}^{2}Z_{in}=I_{2}^{2}Z_{2}+I_{1}^{2}Z_{1}+I_{2}^{2}Z_{2}=\frac{4}{10}P_{in}+\frac{2}{10}P_{in}+\frac{4}{24}P_{in}$$

The power ratio *R*:

(3) $$\frac{I_{2}^{2}Z_{2}}{I_{1}^{2}Z_{1}}=\frac{4}{2}=R=2$$

Solving Equations (2) and (3) gives

$$\begin{array}{c} Z_{1}=200\;\mathrm{ohms}\\ Z_{2}=100\;\mathrm{ohms} \end{array}$$

**Figure 13:**
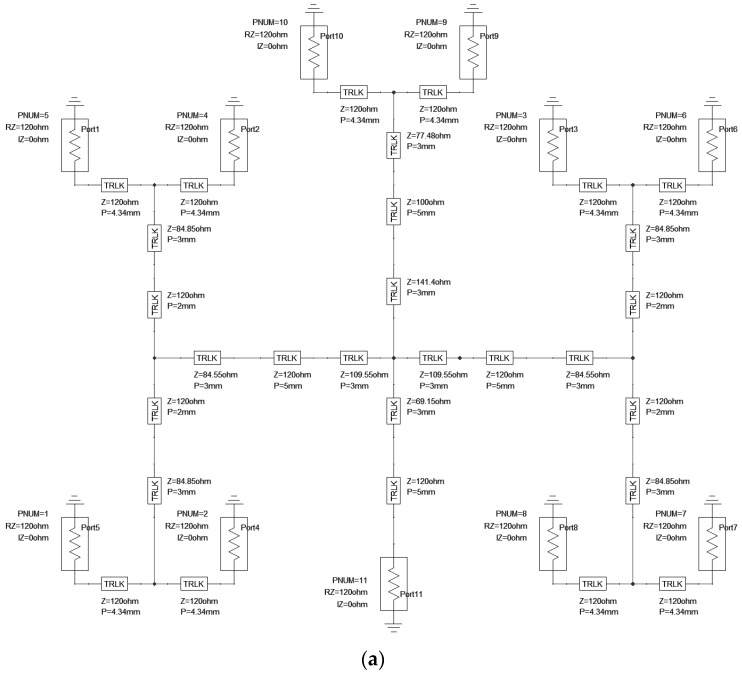

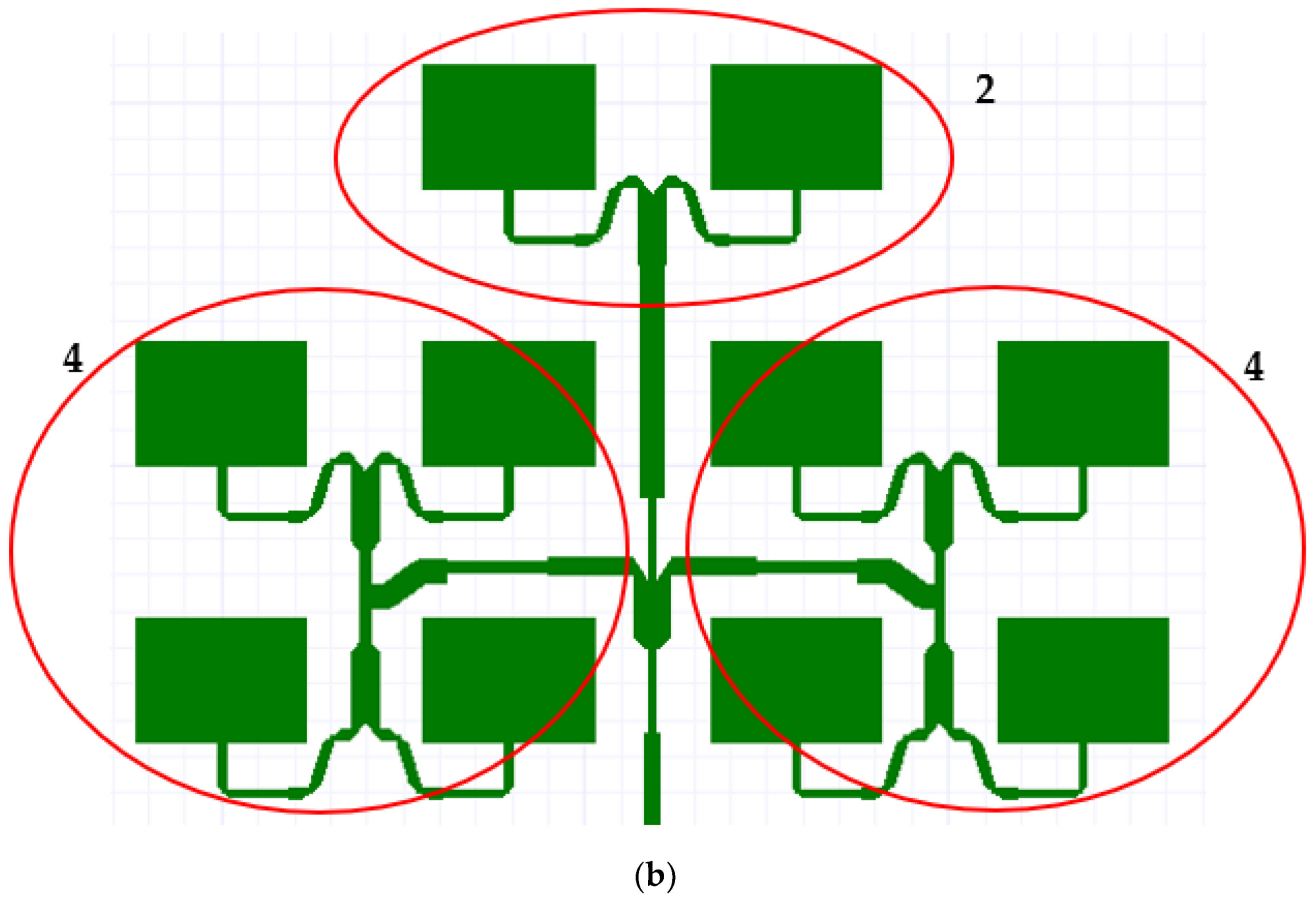
(**a**) Power division network from 1 to 4:2:4 power ratio, with ideal transmission lines. (**b**) Power division network from 1 to 4:2:4 power ratio. Microstrip radiators with microstrip transmission line transformers and the power values are indicated.

For matching, we applied a 141.2 ohms quarter wavelength transformer between 100 ohms, 200 ohms, and 109.55 ohms quarter wavelengths transformer between 120 ohms and 100 ohms (Figure 13a).

The circuit model of the power division network from 1 to 4:2:4 power ratio can be seen in Figure 13a and the layout with the microstrip radiators is shown in Figure 13b.

**Figure 14:**
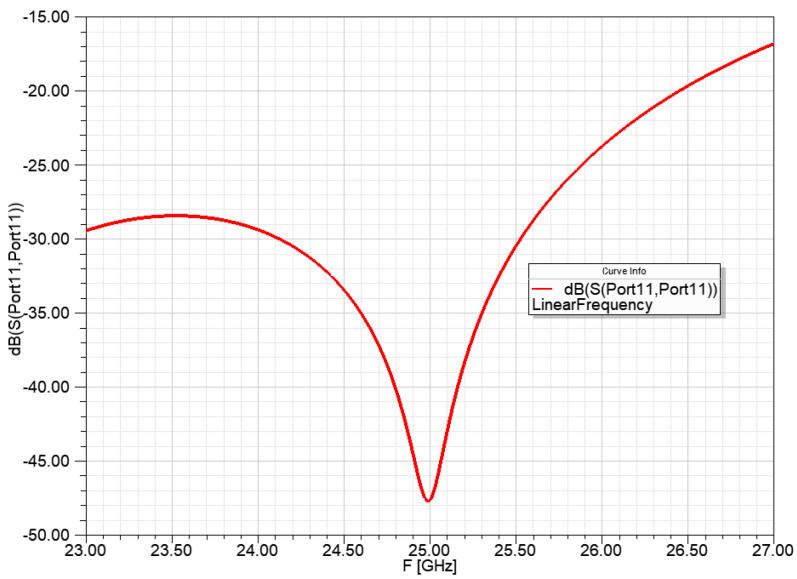
Input reflection of the power division network from 1 to 4:2:4 power ratio, with ideal transmission lines.

The resonance frequency of the power division network for 1 to 4:2:4 power ratio was set to 25 GHz and the input reflection is shown in Figure 14.

**Figure 15:**
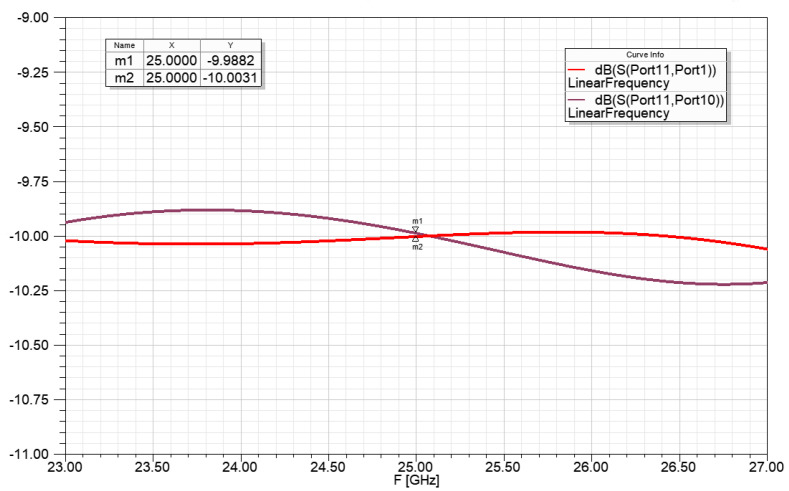
Power ratio for the radiators of the power division network for 1 to 4:2:4 power ratio, with ideal transmission lines.

The Figure 15. Shows the power ratio for the radiators of the power division network from 1 to 4:2:4 power ratio. The −10 dB power transfer means that the network results in a 1:10 power division with less than 0.1 dB power transfer difference in the bandwidth of 24.5–25.5 GHz.

#### 2.3.3. Power Division Network from 1 to 2:10:10:2 Power Ratio

The final part of the feeding network design was the 24 elements’ feeding structure. This power divider is a four ways circuit with a 1 to 2:10:10:2 power ratio.

The design of the power divider was based on two equations, the impedance matching equation, and the power ratio equation.

The equation for impedance matching:

(4) $$\frac{1}{Z_{in}}=\frac{1}{Z_{1}}+\frac{1}{Z_{1}}+\frac{1}{Z_{2}}+\frac{1}{Z_{2}}=\frac{2}{Z_{1}}+\frac{2}{Z_{2}}$$

where

$Z_{in}$ the antenna input impedance, 50 ohms,

$Z_{1}$ the impedance of ways 1 and $Z_{2}$ the impedance of way 2.

The power sum equation:

(5) $$P_{in}={\left[2I_{1}+2I_{2}\right]}^{2}Z_{in}=I_{1}^{2}Z_{1}+I_{2}^{2}Z_{2}+I_{2}^{2}Z_{2}+I_{1}^{2}Z_{1}=\frac{2}{24}P_{in}+\frac{10}{24}P_{in}+\frac{10}{24}P_{in}+\frac{2}{24}P_{in}$$

The power ratio:

(6) $$\frac{I_{2}^{2}Z_{2}}{I_{1}^{2}Z_{1}}=\frac{10}{2}=R=5$$

Solving equations gives

$$\begin{array}{c} Z_{1}=Z_{in}\left(2+2R\right)=600\;\mathrm{ohms}\\ Z_{2}=Z_{in}\left(\frac{4+4R}{2R}\right)=120\;\mathrm{ohms} \end{array}$$

**Figure 16:**
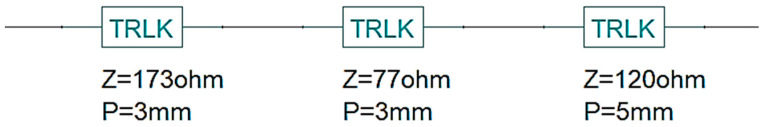
Two-stage quarter wavelength transformers.

The 600 ohms microstrip transmission line is difficult to implement because of its small width, therefore we implemented two-stage quarter wavelength transformers between 120 ohms and 600 ohms. Our technology makes it possible to realize less than 200 ohms of microstrip transmission lines. The two-quarter wavelength transformers’ characteristic impedances are 77 and 173 ohms (Figure 16).

**Figure 17:**
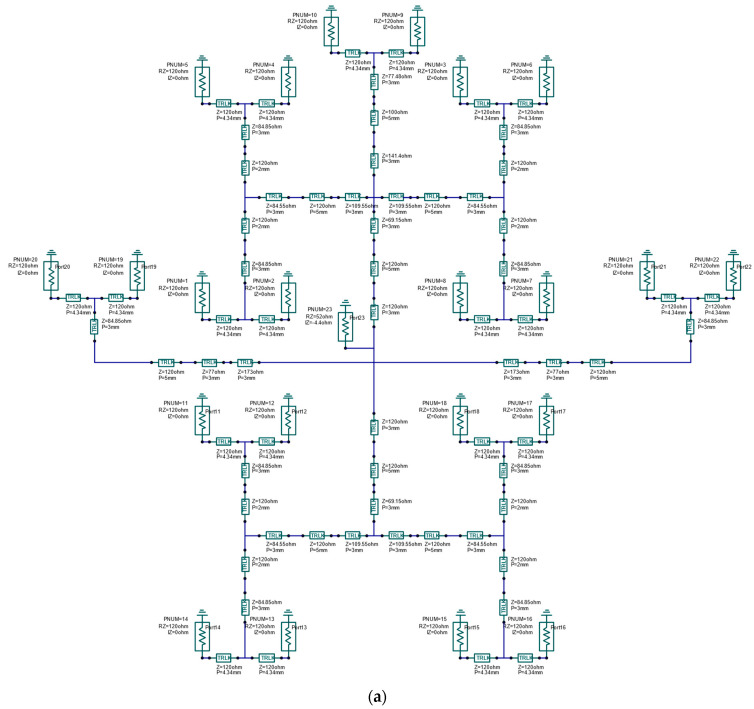

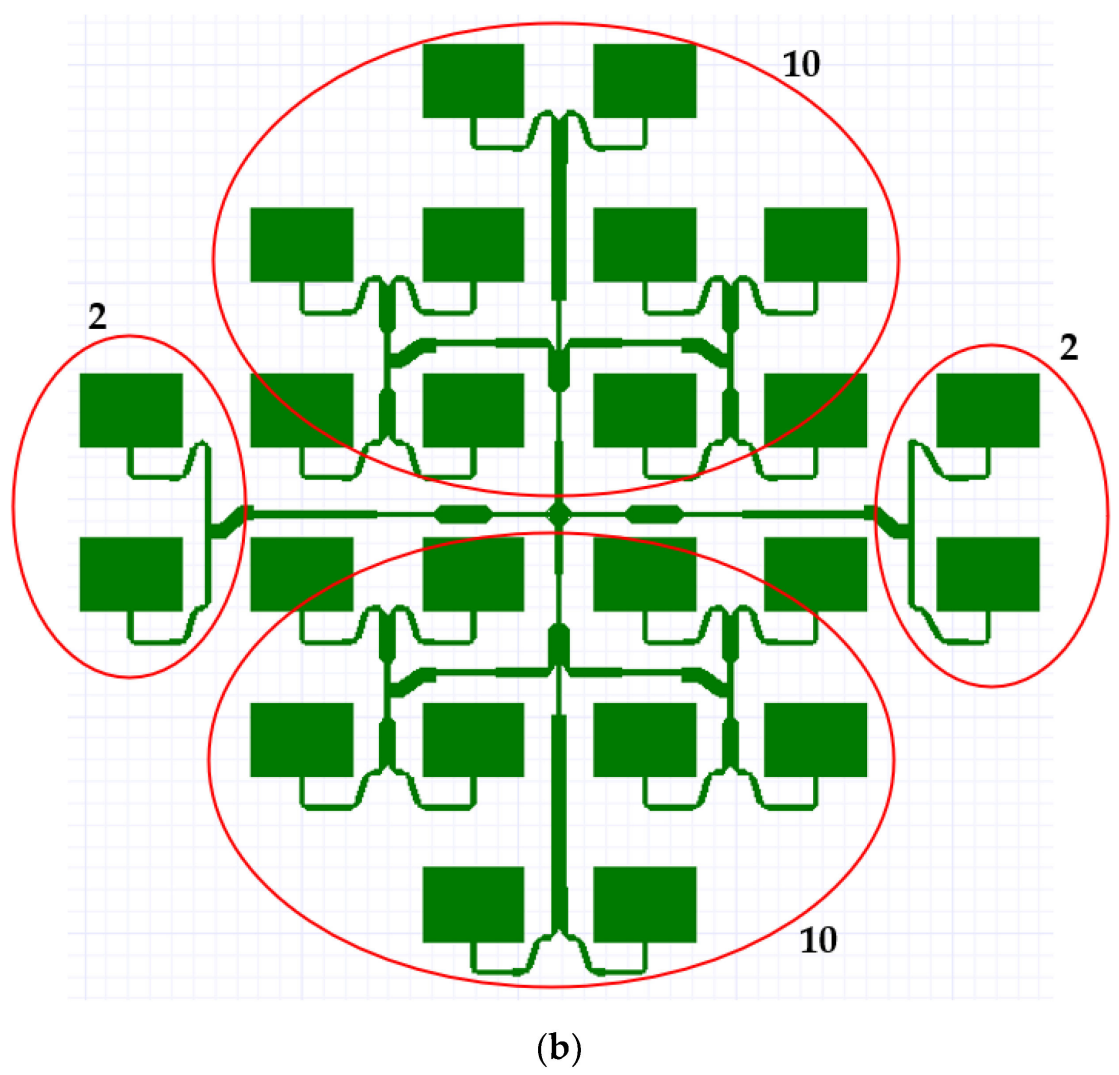
(**a**) Power division network for 1 to 2:10:10:2 power ratio, with ideal transmission lines. (**b**) Power division network for 1 to 2:10:10:2 power ratio. Microstrip radiators with microstrip transmission line transformers and the power values are indicated.

The whole system is symmetrical in the horizontal plane and is completed by an additional 2 × 2 elements left and right (Figure 17).

**Figure 18:**
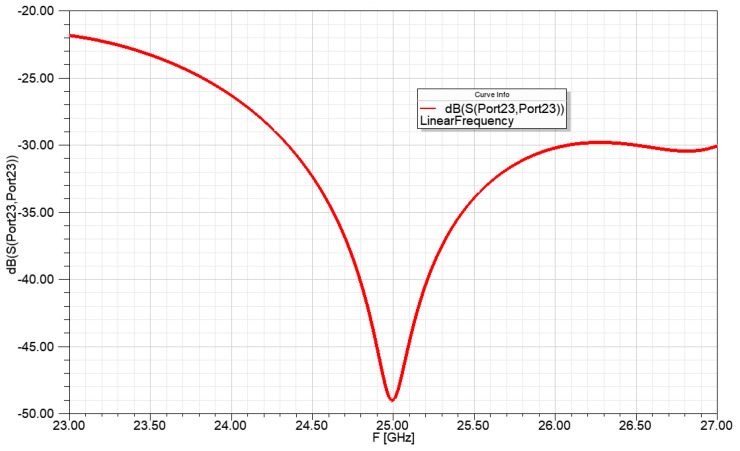
Input reflection of the power division network for 1 to 2:10:10:2 power ratio, with ideal transmission lines.

The resonance frequency of the power division network for 1 to 2:10:10:2 power ratio was set to 25 GHz and the input reflection is shown in Figure 18.

**Figure 19:**
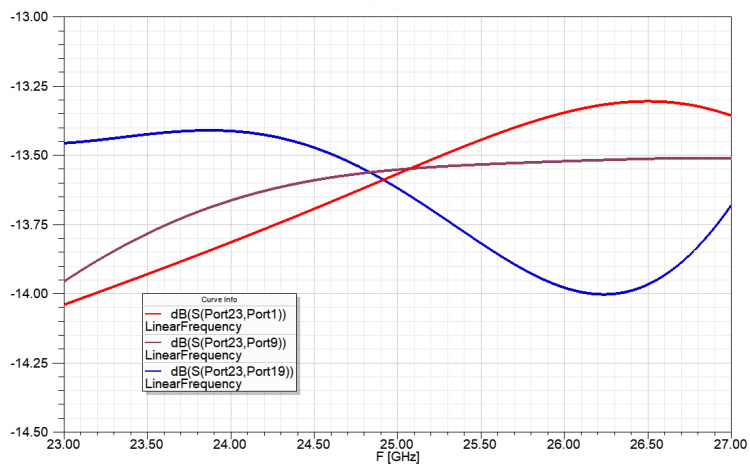
Power ratio for the radiators of the power division network for 1 to 2:10:10:2 power ratio, with ideal transmission lines.

Figure 19 shows the power ratio for the radiators of the power division network from 1 to 2:10:10:2 power ratio. The 1:24 power division with less than 0.15 dB power transfer difference at 25 GHz center frequency and less than 0.35 dB power transfer difference in the bandwidth of 24.5–25.5 GHz.

## 3. Results

After the design and optimization of the feeding network, the CST Microwave Studio was used for the simulation and fine-tuning of the antenna array (Figure 19). Our most important technical parameters were the input reflection bandwidth and the SLL [30,31,32,33].

**Figure 20:**
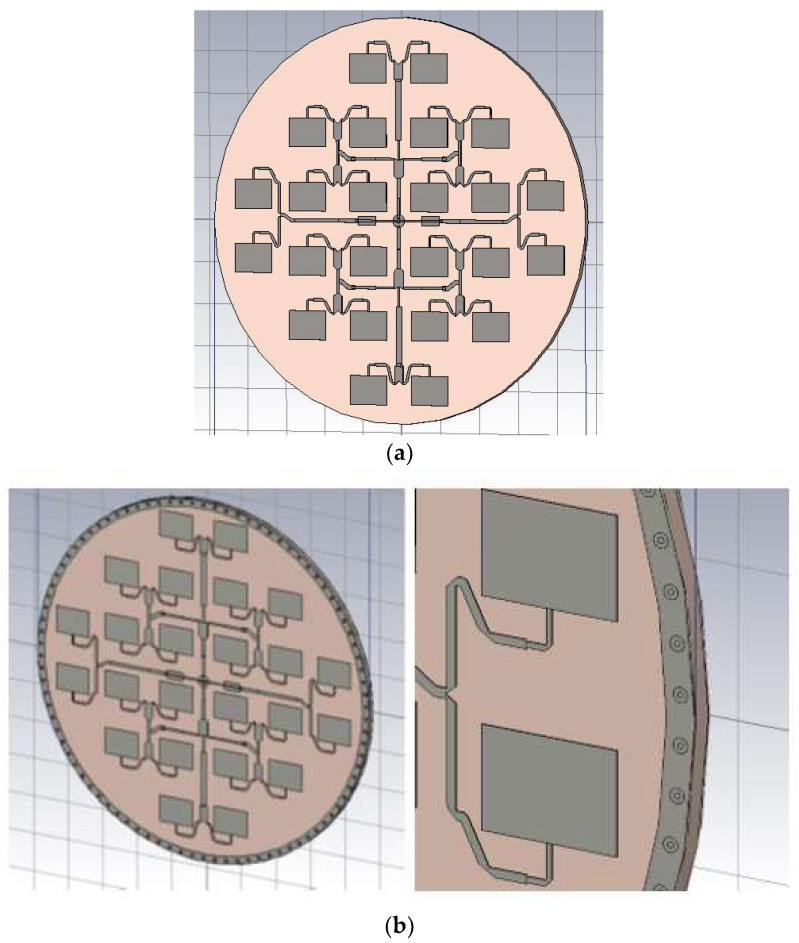
(**a**) CST MWS model of the microstrip antenna array with 24 elements, without SLL suppression ring. (**b**) CST MWS model of the microstrip antenna array with 24 elements, with an SLL suppression ring.

Two versions of antennas were investigated. After the first simulation was found, that the SLL is not acceptable in some directions, therefore an optimized ring with ground vias was implemented, to increase the antenna sidelobe level (SLL) (Figure 20b).

**Figure 21:**
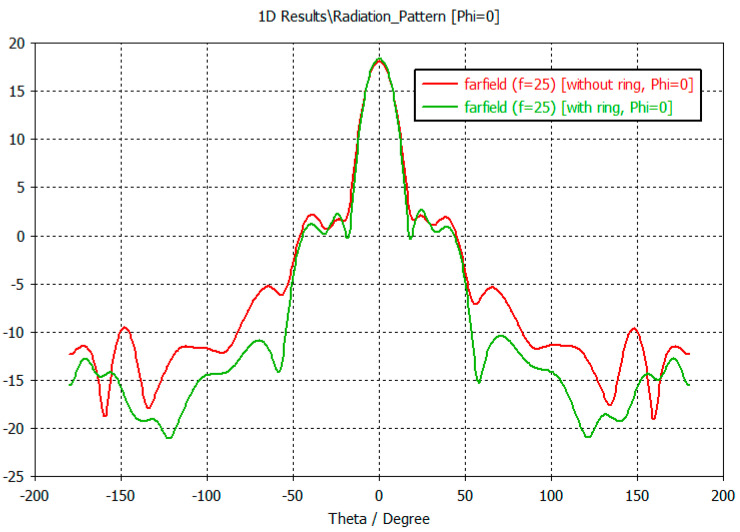
The radiation pattern of the microstrip antenna array with 24 elements, simulated with CST MWS, without, and with an SLL suppression ring (Phi = 0 plane).

**Figure 22:**
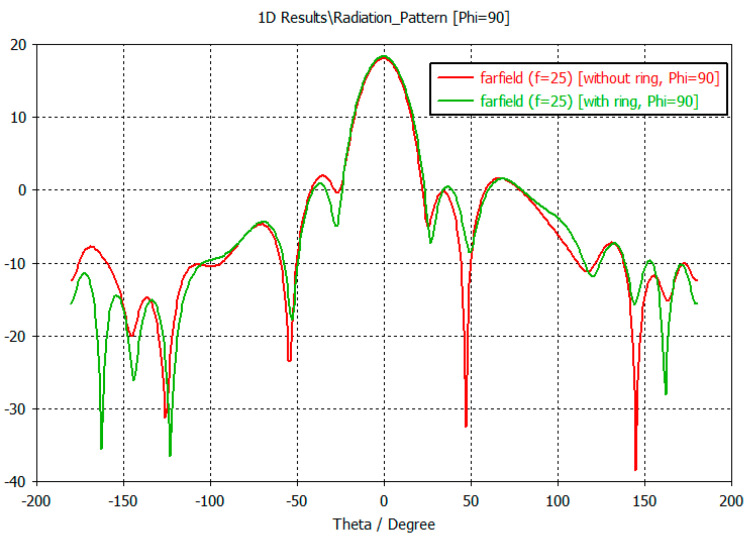
The radiation pattern of the microstrip antenna array with 24 elements, simulated with CST MWS, without, and with an SLL suppression ring (Phi = 90 planes).

Examination of the radiation patterns showed that the SLL could be reduced by up to 5 dB in some directions by using the ring. The antenna gain is not changed significantly. The SLL reduction was significant in the Phi = 0 plane in the elevation angle range of 50–180 degrees (Figure 21). Here the SLL reduction was around 5 dB. On the other plane in the Phi = 90 the SLL reduction can be experienced only at back range (Figure 22).

**Figure 23:**
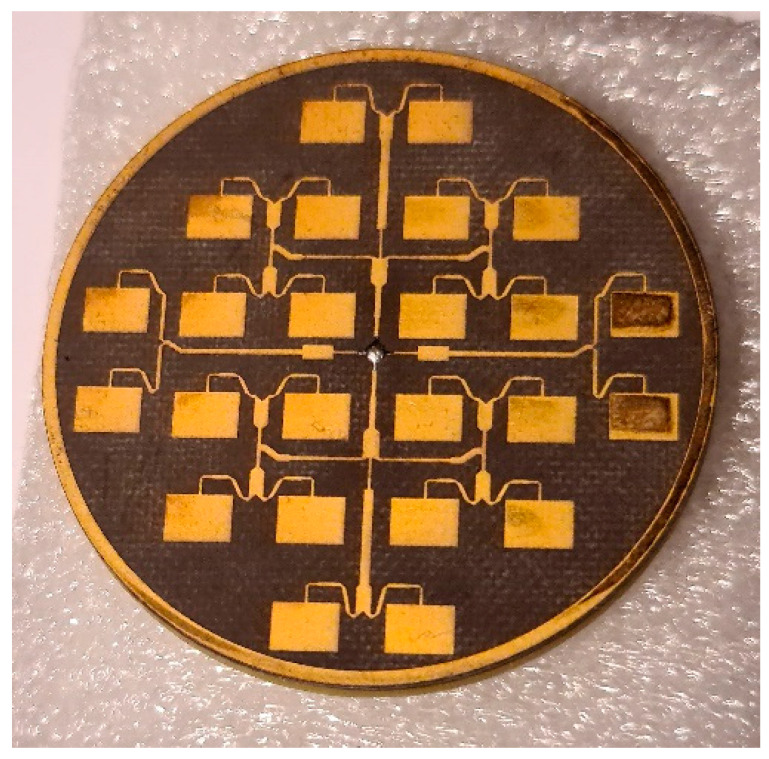
Microstrip antenna array with 24 elements.

After simulation verification of the antenna’s functionality, the antenna was built (Figure 23).

**Figure 24:**
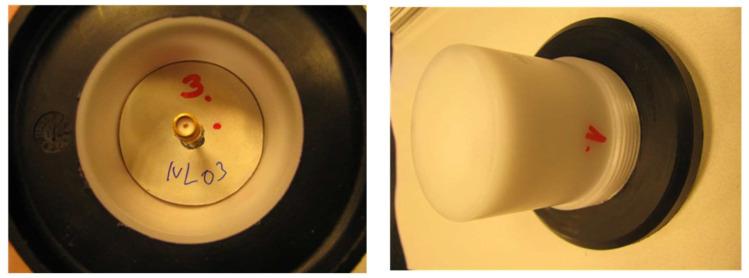
Microstrip antenna connector and radome.

For the measurements, an SMA connector was connected to the antenna input with a semirigid coaxial transmission line, and a radome was applied (Figure 24).

The measurements were performed in the antenna measurement room of the Department of Broadband Infocommunications and Electromagnetic Theory of the Budapest University of Technology.

**Figure 25:**
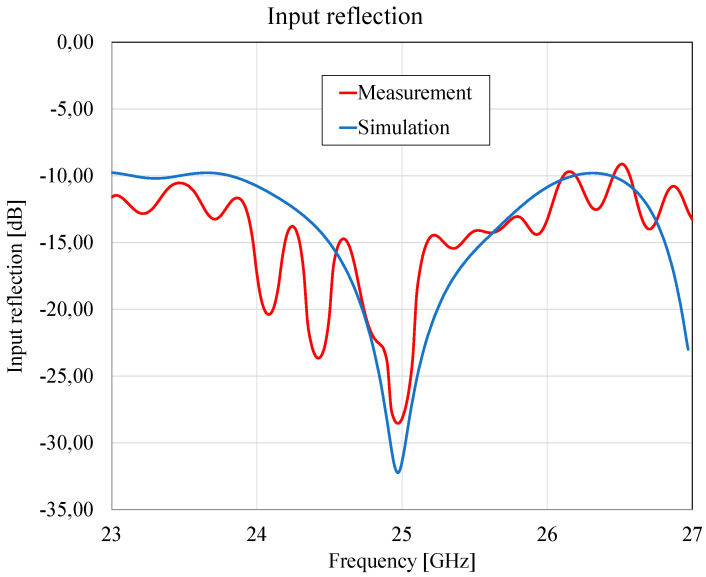
Microstrip antenna input reflection.

The antenna input reflection in the 23–27 GHz frequency band shows a good agreement between simulated and measured values (Figure 25).

**Figure 26:**
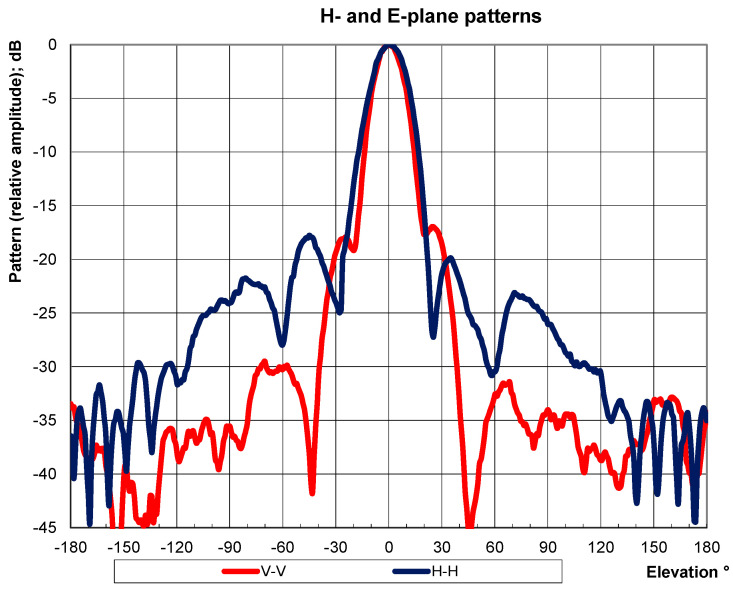
Microstrip antenna measured radiation pattern.

The antenna radiation pattern was measured in the two main planes and the results show SLL better than −17.3 dB (Figure 26).

Finally, we compared and summarized the most important antenna radiation pattern characteristics in Table 3.

It can be concluded that the parameters of the antenna have been measured and verified to meet the specification. The parameters were achieved by using a special feeding network and a sidelobe suppression ring.

## 4. Conclusions and Future Work

We presented a systematic design of a microstrip antenna array and its feeding network. A new antenna layout was proposed for the tank level meter radar sensor. The microstrip antenna elements optimally fill the opening of the tank, therefore maximal gain can be reached. The optimized outer ring ensures the improved sidelobe level to reduce the side reflections from tank sidewalls. The correctness of the design was verified by measurements.

We plan to investigate the antenna further, mainly to improve the feeding network with non-uniform illumination. Another possibility for further development is the use of artificial material as the sidelobe suppression ring.

## Figures and Tables

**Table 1:** Comparison of single MPA on different substrates for Bandwidth and Efficiency.

Substrate	RT5880 ε_r_ = 2.2 tan δ = 0.0009		RO4003 ε_r_ = 3.55 tan δ = 0.0027		RO3010 ε_r_ = 10.2 tan δ = 0.0035	
Height [mils]	BW [%]	Efficiency [%]	BW [%]	Efficiency [%]	BW [%]	Efficiency [%]
5	0.9	96.1	0.8	92.8	0.3	88.7
10	1.9	92.5	1.6	86.6	0.8	79.7
20	4.2	86.1	3.4	76.3	1.7	66.3
31	6.9	80.0	6.2	67.6	3.2	55.9
62	16.8	66.6	16.6	51.0	9.3	38.8

**Table 2:** Comparison of uniform 6 × 6 and 24 elements array.

Array	Gain [dB]	Beamwidth [deg]	SLL [dB]
6 × 6 uniform	22.1	14	−13.7
24 elements	20.6	17.4	−15.8

**Table 3:** Comparison of antenna radiation patterns.

	Gain [dB]	Beamwidth [deg] HH	Beamwidth [deg] VV	SLL [dB]
Simulation	18.4	15	21	−15.7
Measurement	18.0	16	19	−17.3

The main parameters of the antenna are Reflection bandwidth = 2.2 GHz (8.8%), Gain = 18 dB, Sidelobe level = −17.3 dB.
